# Introducing peer worker roles into UK mental health service teams: a qualitative analysis of the organisational benefits and challenges

**DOI:** 10.1186/1472-6963-13-188

**Published:** 2013-05-24

**Authors:** Steve G Gillard, Christine Edwards, Sarah L Gibson, Katherine Owen, Christine Wright

**Affiliations:** 1 St George’s, University of London, London, UK; 2 Kingston University Business School, Kingston, UK

**Keywords:** Mental health, Peer support, Secondary data analysis (qualitative), Health services research, Recovery, Consumer participation, Service user involvement, Community psychiatry, Role adoption, Workforce development

## Abstract

**Background:**

The provision of peer support as a component of mental health care, including the employment of Peer Workers (consumer-providers) by mental health service organisations, is increasingly common internationally. Peer support is strongly advocated as a strategy in a number of UK health and social care policies. Approaches to employing Peer Workers are proliferating. There is evidence to suggest that Peer Worker-based interventions reduce psychiatric inpatient admission and increase service user (consumer) empowerment. In this paper we seek to address a gap in the empirical literature in understanding the organisational challenges and benefits of introducing Peer Worker roles into mental health service teams.

**Methods:**

We report the secondary analysis of qualitative interview data from service users, Peer Workers, non-peer staff and managers of three innovative interventions in a study about mental health self-care. Relevant data was extracted from interviews with 41 participants and subjected to analysis using Grounded Theory techniques. Organisational research literature on role adoption framed the analysis.

**Results:**

Peer Workers were highly valued by mental health teams and service users. Non-peer team members and managers worked hard to introduce Peer Workers into teams. Our cases were projects in development and there was learning from the evolutionary process: in the absence of formal recruitment processes for Peer Workers, differences in expectations of the Peer Worker role can emerge at the selection stage; flexible working arrangements for Peer Workers can have the unintended effect of perpetuating hierarchies within teams; the maintenance of protective practice boundaries through supervision and training can militate against the emergence of a distinctive body of peer practice; lack of consensus around what constitutes peer practice can result in feelings for Peer Workers of inequality, disempowerment, uncertainty about identity and of being under-supported.

**Conclusions:**

This research is indicative of potential benefits for mental health service teams of introducing Peer Worker roles. Analysis also suggests that if the emergence of a distinctive body of peer practice is not adequately considered and supported, as integral to the development of new Peer Worker roles, there is a risk that the potential impact of any emerging role will be constrained and diluted.

## Background

A range of different approaches to employing people with personal experience of mental health problems, specifically to make direct use of that lived experience in supporting others with similar problems (as peers), has been identified internationally [1]. A number of terms including Peer Support Worker, Peer Support Specialist and Consumer-Provider have been used to identify these roles. We will use the generic term Peer Worker throughout this paper because it does not specify or limit the range of roles under consideration while at the same time it makes it clear that we are interested in people intentionally employed to make use of their personal experiences of mental health issues in the delivery of services.

### Peer workers and the evidence base

The evidence base for the impact of mental health service initiatives employing Peer Workers is growing and originates mostly from North America, Australia and New Zealand. Reducing admission to inpatient psychiatric care is the most reported outcome, and has been evaluated through observational [2], comparison group [3] and cross sectional [4] study designs. A before-and-after study [5] and a cross sectional survey [6], both from the US, found significant improvement in individual empowerment associated with receiving peer based support. Hope and strength of social networks have also been indicated as important outcomes for service users (consumers) in receipt of support from Peer Workers [7,8].

A recent literature review [9] details a relative wealth of qualitative research based on lived experience that attests to the benefits of Peer Worker initiatives in mental health services, for Peer Workers themselves and the service users they support. Benefits include enhanced personal sense of empowerment, developing better social support and furthering personal recovery [7,10]. Some of this qualitative literature has also begun to identify organisational challenges to introducing Peer Workers into mental health service teams, including establishing appropriate boundaries [11], power imbalances within teams [12], stress experienced by Peer Workers [13] and the importance of role distinctiveness [11]. It has been suggested that the introduction of Peer Workers represents a unique set of challenges to existing mental health workforce practices, necessitating further research that pays closer attention to the specifics of service context [14] and acknowledges a learning curve in the development of role, the needs of the worker and the workplace environment [15]. An established organisational research literature provides insight into the development and adoption of new work roles, highlighting issues of role distinctiveness, team consensus around role and institutional support as key to the adoption process [16,17]. We use this literature to provide a relevant conceptual underpinning to our analysis.

### Peer workers and UK mental health and social care policy

In UK health and social care policy the introduction of Peer Workers into the mental health workforce is perceived to offer an opportunity to address capability and skill mix in mental health teams [18] and organisational productivity [19]. Peer support has been identified as a key facilitator across a range of UK health and social care policy agendas, including mental health recovery [20], self-care [21] and personalised health and social care [22]. An implementation programme to support the UK mental health strategy [23] has been established in England with a specific remit to develop and demonstrate new Peer Worker roles [24].

#### Aims

The lack of a more established evidence base describing the organisational benefits and challenges of introducing Peer Workers into mental health service teams is a concern given current policy impetus. Mental health service provider organisations and teams would benefit from empirically grounded learning that can be applied in practice. In response to this need for evidence, this paper aims:

1) To describe the emergence of Peer Worker roles in mental health services in England from the perspectives of mental health service users, Peer Workers, and mental health service staff and managers;

2) To describe in detail the organisational benefits and challenges of introducing Peer Worker roles into existing mental health service teams.

#### Study design

This paper reports a secondary analysis of in-depth qualitative interview data. The primary study was a mixed-method organisational study of initiatives supporting self-care in mental health [25]. The aim of the primary study was to identify barriers and facilitators to providing self-care support in Mental Health National Health Service (NHS) Trusts (public sector health service provider). The study included qualitative interviews with 121 service users at two time points, when they first accessed interventions supporting self-care and nine months later. Interviews were comprised of questions about interviewees’ expectations and experiences of support for self-care respectively. Qualitative interviews were also held with 30 staff team members (including Peer Workers, non-peer staff team members and team managers) working in those interventions, exploring their experiences of supporting self-care. In referring to staff team members as ‘non-peer’ we acknowledge that staff in mental health services may have personal experiences of mental health issues, and that those experiences may inform their work. The distinction we intend to make is with staff specifically employed to explicitly and openly make use of those experiences in their work. NHS ethical approval for the study included analysis and reporting of anonymised, verbatim data in subsequent publications.

#### Setting

Four English mental health NHS Trusts in three study sites developed innovative interventions providing support for self-care for mental health service users, all of which included Peer Worker roles as core components of the intervention. Each intervention had different arrangements for employing and training people with personal experiences of mental health issues to work as Peer Workers.

In a London mental health Trust peer support groups for people experiencing personality disorders were co-facilitated by Peer Workers (Support Facilitators) alongside Liaison Workers who were health and social care professionals. Support Facilitators had all used the peer groups in the past and were employed after expressing an interest in the role while using the groups. In an earlier incarnation of the project Peer Workers had been employed as volunteer Lead Service Users and also continued to use the groups. The service was then restructured and Peer Workers became paid employees of the Trust, first on the bank of temporary staff and then as contracted, part-time members of staff. Peer Workers completed an eight session training before taking up the role, and received ongoing training and supervision as part of the job.

In a South of England mental health Trust Peer Workers were employed as trainers supporting service users in developing Wellness Recovery Action Plans® (WRAP®) [26]. Peer Workers were recruited through services in the Trust and were paid part-time employees of the Trust. They undertook WRAP® training themselves and developed their own WRAP® plans before delivering training to a range of service users and Trust staff, either alongside professional trainers or on their own.

Two North of England mental health Trusts worked in partnership with community sector organisations providing community arts projects that aimed to support the social inclusion of mental health service users. A range of arts activities were co-facilitated or led by Peer Workers who had attended the arts projects in the past. Peer Workers were provided training and support by the community organisations and worked for the community organisations as unpaid volunteers or paid part-time employees. Key differences and similarities between the three study sites are indicated in Table 1 below:

**Table 1:** Study sites

**Site**	**Peer Worker role**	**Aim of the service**	**Recruitment approach**	**Terms and conditions**	**Training**	**Ongoing support**
**London**	Group co-facilitator (community-based peer support groups for people experiencing personality disorders)	To build empowerment; develop coping strategies	Informally, as group member	Initially, voluntary; subsequently, paid part-time member of Trust staff	Eight session training in supporting group therapeutic process	Regular formal supervision; ongoing training with staff team
**South**	Wellness Recovery Action Planning® (WRAP®) Trainer for people using mental health services	To enhance personal recovery and self-care skills	Informally, as completer of WRAP® training	Paid part-time member of Trust staff	Completion of own WRAP® training (seven sessions); brief initial training	Regular formal supervision
**North**	Group co-facilitator (community arts projects for people using mental health services)	To support social inclusion	Informally, as group member	Unpaid volunteer or paid part-time employee of arts organisation	Brief initial training	Informal one to one support as required

#### Sample

The data reported and analysed in this paper were collected from 41 participants in the main study from across sites and across stakeholder groups; Peer Workers, their managers and the non-peer staff they worked alongside, and the service users to whom they provided a service. As a secondary analysis process, data rather than participants were selected for inclusion in the analysis (see Methods section below). Some participants – typically Peer Workers, managers and non-peer staff – talked extensively in interview about their experiences of being a Peer Worker or working with Peer Workers respectively, and so large amounts of data are included from those participants. Other participants only talked briefly about Peer Workers, particularly service users, many of whom only spoke about their decision not to become a Peer Worker. We reflect on this imbalance in the data in the Discussion section. Characteristics of participants are shown in Table 2 below, indicating the role and site of each individual:

**Table 2:** Data sources

	**Managers**	**Peer Workers**	**Non**-**peer staff**	**Service users**	**Total** (**sites**)
**London**	2	3	1	9	**15**
**South**	1	6	4	3	**14**
**North**	1	6	4	1	**12**
**Total** (**roles**)	**4**	**15**	**9**	**13**	**41**

## Methods

In our original analysis of qualitative interviews we coded interview data, using qualitative analysis software, to a range of themes as part of the process of understanding how mental health self-care was supported by UK mental health NHS Trusts [25]. Interview schedules had been designed to elicit data on support for self-care, and many interviewees spoke about Peer Worker roles during the course of the interviews. A number of the themes that emerged in our original analysis were relevant to the introduction of Peer Worker roles: ‘new roles and relationships’; ‘peer support’; ‘service users as staff’. In the secondary analysis we collated data coded to those three themes and subjected that data to a secondary thematic analysis using analytical processes informed by Grounded Theory. Grounded Theory is an appropriate approach where there is little formal knowledge about a phenomenon – in our case, the organisational processes of introducing Peer Worker roles into mental health services – and where there is data of sufficient richness to enable the development of theoretical or conceptual understandings grounded in that data [27].

The secondary analysis was undertaken in five stages. First, the first author (SGG) completed a preliminary, open coding of the data collated for this analysis. Language from interview transcripts was used where possible in generating codes. Second, the first author developed a preliminary set of data categories that grouped together codes related in content and meaning. Third, the first author coded the whole data set to these categories using a process of constant comparison [27], refining category content and boundaries where necessary rather than fitting data to preliminary categories. The first three stages of the analysis process produced a provisional set of twelve categories.

The fourth stage of the process was designed to improve the methodological rigour of the analysis process through checking the validity of categories and the reliability of coding data to those categories. Two other researchers (SLG, KO) independently coded the first fifty sections of text coded by SG in stage three to the same set of categories. Data were coded to more than one category where researchers felt there was multiple fit, and not coded where they did not feel that there was an appropriate category. The researchers also made notes about the content, boundaries and appropriateness of categories.

The two additional researchers had not been part of the primary study team but were experienced service user researchers working with SG on a subsequent organisational study about Peer Workers in mental health services. As current or former users of mental health services the additional researchers brought a different standpoint to the analysis processes than did the first author; all three researchers had extensive experience of university-based mental health research. Through this process we sought methodological rigour not just as a simple test of reliability in the coding process but also as a means of understanding how researchers of different standpoints might interpret the data differently, thus enriching the analysis. Other work by members of the team has demonstrated how researchers of different standpoints can produce different ‘analytical narratives’ on the same dataset [28] and how as teams we coproduce our analysis through discourse within the team [29].

The two researchers’ coding was then checked against the first author’s coding for agreement. Researchers were considered to be in agreement where they coded a section of text to at least one category in common. The first author was in agreement with one researcher – KO – on 74% of the 50 sections of coded text, and with the other researcher – SLG – on 70% of coded text. All three researchers were in agreement on 56% of text coded. These levels of agreement were lower than the findings of a similar process [30] that reported four researchers agreeing on 65.5% of codes assigned to 39 sections of text (with three of the four researchers agreeing on 84% of codes). We decided that there was sufficient lack of consensus in the coding in this study to necessitate revision of the set of categories. Based on notes made by the additional researchers and on subsequent discussion we produced a refined set of eight categories. A number of new codes were suggested by the additional researchers that added meaning and complexity to the content of each category.

The final stage of the analysis involved moving beyond this set of descriptive categories and developing a number of focused themes [31] that would meaningfully address our research aim of identifying the organisational benefits and challenges of introducing Peer Worker roles in mental health services. We did this through an iterative process of looking for relationships in our data that had meaning across data categories, moving back and forth between those emerging thematic relationships and our dataset as a whole in a further process of constant comparison [27]. This process was led by the first author with feedback from the other authors until a focused set of themes was agreed. We use the role adoption literature we refer to elucidate those emerging themes in the Discussion section.

## Results

A set of five focussed themes was developed, as described above, and these are presented in the commentary that follows. The sets of codes, categories and themes produced through this process are illustrated in (Additional file 1: Table S3) below, indicating how themes ‘cut across’ our categorical organisation of the data:

As well as fulfilling our first aim – to describe the emergence of Peer Worker roles in mental health services from a range of stakeholder perspectives – the five themes also serve our second aim; to develop understanding at an organisational level of some of the benefits and challenges around introducing Peer Worker roles. We begin by exploring a range of issues around the recruitment process and how potential Peer Workers were identified in our cases. Once recruited, our provider organisations had to work on integrating new Peer Workers into existing mental health services teams, and that experience impacted on identity for the individual Peer Workers involved. In all our study sites the introduction of Peer Workers into team challenged existing practice boundaries. Finally we consider the extent to which a distinctive body of practice was emerging in the work that Peer Workers were doing. Our analysis attempts to develop understandings of these processes and dynamics that are informative for other mental health providers and teams as they develop and introduce new Peer Worker roles.

### Who becomes a peer worker, how and why?

This theme emerged from a large body of data around employment issues and the perceived benefits of working as a Peer Worker. In all our cases Peer Workers were recruited directly from the service they would be working in through an informal or semi-formal process; staff working in the service approached service users who they thought might be appropriate and asked them if they were interested in taking on the role. In the South of England case past experience of using the service was widely seen as an asset to taking on the Peer Worker role:

It's actually very similar to being part of the group because it is still about sharing experiences and giving examples. It's just that rather than being asked for those examples … it is more a case of ‘this is how it is, this is an example, how does that, does that gel with you?’ (South Peer Worker)

In all cases Peer Workers’ motivations for taking on the role were largely around using their own, often difficult experiences of mental illness to help others with similar experiences:

If the young people want to talk to me my ears are there, my shoulder is there and everything they say is confidential and I would try to help them the best I can as well as what I’ve had done to me really, helped been supported. (North Peer Worker)

I have empathy with people and I felt I can give. (London Peer Worker)

In contrast, managers in all sites spoke about the benefits to the individual Peer Worker of the role as a developmental opportunity, and particularly as a route to further employment. In each case the potential benefit to Peer Workers was an important rationale for employing service users in Peer Worker roles:

It’s good for the support, people want to become support workers, it gives them experience of working with people, it gives them group facilitation skills, personal development skills, self confidence, self esteem and probably, so it's very good for them. (North manager)

This data is suggestive of a tension between the motivation of recruiting managers, at least in part pre-selecting potential Peer Workers on the basis of their likelihood to benefit personally, and the motivation of service users to work as Peer Workers on the basis of sharing their personal experiences with other service users. This tension is explored further in the data analysis that follows.

Once approached about becoming a Peer Worker, service users weighed up the pros and cons of taking on the role. Some London service users were put off from taking on a Peer Worker role because the issues they would have to deal with were too close to their own experiences. It should be noted that the group in London often supported service users experiencing severe crises:

With this it felt more sensitive, it felt more close to the bone. (London service user)

It might not suit me, like I’m trying to get away from mental health stuff … I’m doing quite well at the moment. (London Service User)

In London a manager and Peer Workers also queried whether prior experience of using the service was sufficient criteria to work as a Peer Worker:

It was designed to be self-selective so that people could come along and you know it’s a very difficult thing, what you think you can do and what you can actually can manage, they can be two very different things … people think ‘actually I really can’t do this work, I am really not willing to go this far.’ (London manager)

We put ourselves forward and then it’s processed by the management, and because we’ve been in the groups, you meet the criteria. (London Peer Worker)

A range of interviewees reflected on the importance of being ready to take on a Peer Worker role:

There was a time when I wasn’t ready … I’m more flexible, I’ve learned to be more flexible. (South Peer Worker)

No. That would be something way, way in the future. You can do that but you have to be ready. (London service user)

In London Peer Workers and a manager reported drop out during training or early into the post among Peer Workers who found the role more demanding than they had anticipated:

Some members started and they found out it was too difficult for whatever reason, so we lost a few you know, initially (London Peer Worker)

We started off with a group of nine … At the end of that training we had five people and then two months later the number dropped and we were down to four people and then those people were the ones who said “listen, I find it really difficult, I am finding it difficult to manage this working in the groups” and so then they progressed. (London manager)

This data is indicative of possible limitations in informal or semi-formal recruitment approaches. Where the rationale for working as a Peer Worker might be different for recruiting manager and potential Peer Worker, individuals might be selected who are not best placed either to benefit personally or to deliver benefits to the service. In addition, in a service that is clinically demanding – such as the personality disorders project in London – the role might be particularly challenging if clinical issues are close to the Peer Worker’s own experience.

### Building new teams

A further body of data, focused on the Peer Worker/ non-peer staff relationship, alludes in detail to processes of building new teams that incorporate both peer and non-peer staff roles. Staff and managers spoke positively about their new relationship with former service users and felt there was equality in the relationship:

I’m working alongside those individuals as my colleagues so I’m not … working with them in any other capacity other than on an equal footing … it’s not any different. (South staff)

Difficulties implicit in the changing relationship were acknowledged:

You have to be on your best behaviour, because if you get upset, if I get upset I’m worried that people are going to think, ‘ooh, she’s having a service user moment’. There’s lot of pressures to kind of gain acceptance. (London Peer Worker)

… suddenly they’re a colleague and you get to hear about things that you weren’t privy to before and that feels odd and needs time to sort of settle in, to manage. (London manager)

For Peer Workers these difficulties in renegotiating relationships seemed to impact directly on their experiences of joining existing teams. Peer Workers did not share the view that they were equals in the new team:

I wouldn’t say that I feel completely like I’m a member of the team … I guess I’d still feel somewhat inferior in, I don’t know, how I’m seen by the team or by members. (London Peer Worker)

Some staff identified resistance in the existing workforce to the introduction of Peer Workers into the team:

At first some of the staff were quite negative you know. ‘Oh we can’t do that, what if he becomes ill?’ … but now they’re used to him and see that he's a very good worker and that he works really well and everybody's very fond of him. We all get on very well so it’s worked out. (North staff)

It was recognised that the source of this resistance might lie in the training and background of existing staff; a sense that their roles and responsibilities might be threatened by a new Peer Worker role:

You always have a very small minority, as well, that get quite defensive about their roles, and their responsibilities and their occupation, and, well ‘are you saying that we’re not doing it right?’ (South staff)

The flexible terms and conditions of employment offered to Peer Workers were often appreciated, enabling Peer Workers to work when they felt well and reducing experiences of pressure resulting from the role. However some Peer Workers felt that those terms and conditions devalued the role and contributed to a sense of hierarchy:

I think there’s aspects of the role that are really frustrating, such as being on bank staff is really hard sometimes … having a group cancelled just feels crap sometimes … because I’m a co-facilitator, if my co-worker is on leave and the group is cancelled, I don’t work, which means I have less money. (London Peer Worker)

There is a hierarchy in terms of payment and position. (South Peer Worker)

In the London site Peer Workers could feel disempowered by the working arrangements:

You end up feeling powerless a lot of the time because of how things are set up. (London Peer Worker)

The step-up, step-down was supposed to be empowering to people, so they could stand down when they didn’t feel able to work. But on a couple of occasions it’s been sort of used to tell us not to come in, so that’s sort of really difficult. (London Peer Worker)

Again in the London case – where work was more clinically based – there seemed to be a mismatch in expectations. Peer Workers’ lack of specific clinical training and knowledge meant they found it difficult to participate fully in team meetings or to take on the role in group work they had hoped to. This experience was also acknowledged by a non-peer member of the team:

If I’m in that meeting and I say something that isn’t, sort of psychodynamic, it might be a kind of practical idea, then … it’s never like criticized, but I just feel that, it’s not the way things are done. (London Peer Worker)

The initial idea of Lead Service Users taking the groups was so they, members would be even more empowered, really, and run their own groups, but that didn’t kind of happen … when the service started up, it was organized around a clinical model, a kind of clinical direction, being led by a doctor, and the team meetings had a sort of psychodynamic basis, so I think there were certain norms established that would have made it difficult to become what it intended to be. (London staff)

### Being a peer worker: an experience of conflicted identity

Not quite being able to feel part of the staff team also seemed to be reflected in a conflicted sense of identify reported by many Peer Workers. There was consensus among both managers and Peer Workers that the Peer Worker role did offer an alternative, positive identity that was focused on something other than mental ill health:

It’s very powerful how it lifts people out of that sick role, to say, ‘let us give them a job, here’s some responsibility, I believe in you, you can do this’… (South manager)

It’s almost made me feel normal in inverted commas, it’s been a big thing, a great positive and I really look forward to going every time. (North Peer Worker)

However, both Peer Workers and their non-peer colleagues acknowledged that the Peer Worker identity was complex, noting that the role enacted neither a wholly service user-, nor wholly staff identity:

It’s quite a hard place to have that middle ground and being not quite professional, not a service user. (London Peer Worker)

Particularly when you’ve got a service user running the group with you as well, they’re in this sort of ‘no-man’s-land’. They’re not quite staff, they are staff, but they’re not quite the same, because you can’t share certain information with the service users that you might with other staff. (South staff)

The transition at the London site between informal and formal roles provided an opportunity to explore how the challenges of a conflicted identity can be exacerbated by the structuring of the role:

This idea of Lead Service Users as somebody who could … be a member of the group one day and the next day working in the group, created enormous conflict for them … (London manager)

We were meant to be users and stepping up and then stepping down in our groups … other members found doing the same role that they felt intimidated by the members knowing what was wrong with them and they’d seen them in vulnerable situations. (London Peer Worker)

### Challenging boundaries

Peer Workers, managers and non-peer staff all made specific reference to the importance of boundaries both within the team, and between Peer Workers and service users. In all cases there was a perception that the introduction of Peer Workers into existing teams had challenged the boundaries those teams had been used to working within:

We were being with a client and [the Peer Worker] would just burst in and sit down and start listening to what was going on which was very confidential and everyone was getting very upset and angry with him. (North staff)

I think the relationships have to change because, although I don’t see the role of the facilitator as the same as a traditional health care professional, there are still boundaries, and the boundaries are there for people to sort of come up against and give limits. (London Peer Worker)

The need to establish and maintain boundaries seemed in large part to stem from awareness within teams of the mental health needs of new Peer Workers, and that they retained a duty to support Peer Workers with their mental health:

When we first employed our member of staff, he wasn't really complying with his medication. We didn't realise at the time and [he] was becoming quite ill and quite psychotic at times … but we kind of missed the early signs and we all felt terrible because we should have known, working in mental health, about what was happening to him … (North staff)

… the sort of support and supervision they need is actually different to other members of the team, and the flexibility, or working with somebody who may have their own health crises for one reason or another, needing more support. (London manager)

As a result the response from teams to boundary issues was often protective in nature, putting measures in place that would reduce the exposure of the Peer Worker to situations where professional and social contact might overlap:

There has just been written a service user policy for when they’re in that no-man’s-land, because I know they’ve been giving out their phone numbers and meeting, and it’s been quite difficult. (South staff)

Any referrals we get from this particular area we don't pass on to this member of staff … because he has friends amongst the service users in this particular area … he goes out socially with them and so that would have made relationships a bit difficult really. You know if someone was going through problems they might not have felt they could talk to him. (North staff)

Supervision and training for Peer Workers was described as further reinforcing boundaries:

We offer supervision to people and again a lot of these issues can be addressed and you know we have to put in boundaries about confidentiality. (North staff)

You may be asked by a member ‘where do you live?’ or ‘have you taken this medication then?’ … role playing deals with these boundary issues, what to do if you’re asked a question like that, role playing different ways in which to respond and deal with this. (London Peer Worker)

This culture of ‘being boundaried’ seemed to have been absorbed by Peer Workers in the London case, who raised the issue of being boundaried in their work with service users:

I do have to remind myself of the new role. I might be asked by a member ‘where do you live?’, or ‘have you taken this medication then?’ I have to be boundaried for them and for me. (London Peer Worker)

… having to have boundaries with service users is like, different. Especially when … someone shows sort of sexual interest in you, or too much attention, or pats you on the back or touches you in some way, and you think ‘well they can’t really do that’ … it’s difficult to try and sort of tell people that because it kind of distances me more from being a service user, which is good I guess, in doing the job … it’s about safety, your own as well as the group member’s safety. (London Peer Worker)

For some Peer Workers this sense of distance and boundaried conduct was associated with professionalism:

There is a degree obviously to which you have to be professional … how you conduct yourself … professional conduct is something that is covered in the training … I do feel like a professional when I’m up there. (South Peer Worker)

This man who was a patient … said something to me before quite sexist about a certain part of my body … and I had to bite my lip … I don’t know if that’s like a normal thing that people who work here think, they develop a thick skin. (London Peer Worker)

This data raises a question about the extent to which a protective approach to boundary setting within the team might, in creating this ‘safe distance’ between the Peer Worker and service user, inhibit the Peer Worker in giving of their personal experiences of mental health issues in supporting service users. As noted above, this was often Peer Workers’ motivation for taking on the role and we explore below the extent to which this ‘giving of personal experience’ was seen as a component of the emerging practice of Peer Workers.

### Is a body of peer practice emerging?

Our study collected a large body of data on the perceived benefits to the service and the staff team of the introduction of the Peer Worker role. Both Peer Workers and non-peer staff said that they thought that having someone on the team who had experienced the service at first hand helped to engage service users and to role model progress:

It was very useful to be able to have service users who have actually used it and are really able to sell it to you. (South staff)

I’ve been there, done that and bought the t-shirt so I want to put my experience through to them and how to lead them into that right direction instead of going downhill like I did. (North Peer Worker)

It gives us role models for other people that you know people can do it and can move on and progress. (North manager)

Non-peer staff, managers and service users all thought Peer Workers brought insight to the team that would otherwise be lacking:

We sometimes feel as if, ‘are we imposing things on people and being too patronising with the decisions we've made and the groups we're operating?’ … but having a service user on the staff brings us down to earth a bit and kind of opens our eyes a bit more because when you're working you have wonderful ideas of what's good for people, but that might not be the reality of it and we can check it out with our [peer] member of staff. ‘What do you think? Do you, as a service user as well, do you find that a bit patronising or a bit too much or what do you think?’ (North staff)

She was obviously a lot more like tuned in about the structure of the groups and the way that it should go. (London service user)

Managers also identified that Peer Workers brought additional skills and resources to the team:

It makes service users feel comfortable, it makes us approachable, makes it relaxed, makes it safe … You're drawing from a larger resource pool of skills as well because your staff, if it's service users as well so it's unlimited really. (North manager)

This data is suggestive of an emerging body of practice characterising the Peer Worker role, incorporating a number of key elements: demonstration or role modelling of personal recovery to current service users; bringing insight and knowledge to the staff team (enhancing the team’s skills mix); creating a more engaging, relaxed environment that feels safe and is conducive to talking and listening. However, the ‘giving of personal experience’ that Peer Workers envisaged when they took on the role seemed to remain problematic in the context of the boundaried role that we described above:

‘… it has been quite hard to assert myself sometimes and, you know, try and be a professional.’ (London Peer Worker)

In the London case Peer Workers articulated the personal costs of that giving:

Well as a user and being a staff, the risks are that certain things are brought up to you at times. You know, something that can be very painful. (London Peer Worker)

I guess I’m trying to … look out for my own needs … it just ends up feeling worse if you feel that you’re giving, giving, giving and not really getting things back. (London Peer Worker)

At the same time there was a sense that the support and training that was on offer did not always help Peer Workers deal with these issues:

There’s the clinical supervision, to sort out things like boundaries with members and what happens in groups, and there’s management supervision to sort out stuff like time sheets and hours but that doesn’t, it feels like banging your head against a wall sometimes … (London Peer Worker)

There have been ideas maybe to do you know, like an introduction to group analysis, or something, but nothing kind of, nothing beyond that really. I haven’t sort of undertaken any formal training to be a group facilitator. (London Peer Worker)

Again this data speaks of tensions; between what Peer Workers were expected to do (the practice that was valued), and what ‘in practice’ they actually did. The Peer Workers we interviewed wanted to give of their personal experience in supporting others, recognised the difficulties and costs of doing this and felt that they were not always supported to do so. The managers and non-peer staff recognised and valued a number of assets Peer Workers brought to the team, but this did not seem to include the ‘giving of personal experience’. Indeed there was a sense that to do so crossed boundaries that were carefully established to protect Peer Workers and service users. This apparent conflict between ‘giving of personal experience’ as a Peer Worker and ‘trying to be a professional’ (to maintain the prescribed, boundaried role) seemed to encapsulate the tensions inherent in the Peer Worker role and to constrain the emergence of a distinctive body of Peer Practice that the whole team could agree on.

## Discussion

The data presented above describes the introduction of Peer Worker roles into a number of mental health services in England. While reflecting much of what has been observed internationally about the introduction of Peer Worker roles [7,9,13], our analysis was further suggestive of a number of challenges to the development and operationalisation of the role. Organisational research literature on the development and adoption of new work roles [16,17] informed our analysis process, offering a conceptual underpinning that enables us to bring insight to the introduction of Peer Worker roles into mental health services more generally. We discuss our themes as presented above in the context of this literature, as well as other emerging research on Peer Worker roles in mental health services.

In this study we had the opportunity to explore the introduction of Peer Workers at an early, often experimental stage in the developmental process as teams learnt from the experience. Peer Workers were recruited though informal, or semi-formal processes, directly from the service itself in all our study sites. Reflecting other recent findings, service users’ decision making about whether or not to take on the role was at least in part shaped by how ‘ready’ they felt [32]. Existing Peer Workers had been motivated to take on the role as an opportunity to use their personal experiences to help others who shared similar problems, although managers sought to recruit those individuals who they thought would benefit most from employment as a developmental or vocational opportunity. Research into new non-professionally qualified support roles in education and social work has suggested that these roles can either become a first step on new career pathways where the work is worthwhile and satisfying, or employment ‘ghettos’ where new workers find their role to be a repository of professionals’ unwanted tasks [33,34]. Lack of a clear job description has been shown to result in allocation of task outside of the job role as perceived by Peer Workers [35]. While we did not find evidence of Peer Workers being asked to undertake low value tasks, our analysis does suggest that clarity of expectation at the point of recruitment is crucial, and that the informal or semi-formal recruitment processes in place in our cases did not facilitate shared expectation.

We did find evidence that the introduction of Peer Workers into existing teams was challenging. Non-peer staff told us of some professional resistance among colleagues towards the introduction of a new role. Resistance to workforce change and defence of professional jurisdiction has been noted more generally in the organisational literature [36,37], while nursing research has indicated concerns among professionals about dilution to the skills mix [38] and impact on quality of care [39] where non-professionally qualified roles have been introduced into existing teams.

Our evidence suggested that professional resistance was not the main barrier to the introduction of Peer Workers into teams. As has been suggested elsewhere, the flexibility built into Peer Worker roles to offer reasonable adjustments to working conditions also worked to maintain hierarchies within teams [12] and could result in Peer Workers feeling disempowered. We also found, as have others [11,40], that non-peer team members and managers emphasised the importance of management of boundaries, reinforced through training and supervision, primarily to protect Peer Workers themselves and enable them to maintain good mental health in the workplace. As such our analysis suggests that the non-peer team exercised a benevolent power over their Peer Worker colleagues that might constrained the extent to which Peer Workers felt able to engage closely with service users in their work, so impacting on the potential distinctiveness of Peer Worker roles. The generic role change literature emphasises the importance of this distinctiveness if new roles are to be successfully adopted [17], observations echoed in other research into mental health Peer Worker roles [11].

Peer Workers indicated that they took on the role because they wanted to use their personal experiences of mental health issues directly in support of others. Non-peer team members and managers valued Peer Workers as role models for service users, in bringing fresh insight into the team and in helping to create an engaging and comfortable environment. Consensus on role expectation has also been shown to enable the meaningful combination of roles within teams [41], while role conflict and confusion has been identified as undermining the integration of new support roles into existing mental health service teams [42]. Institutional support, including appropriate training and supervision, has been identified as another important facilitator of role adoption [17]. Our analysis suggests that, perhaps as a result of this lack of consensus and/ or a perceived lack of support, Peer Workers felt inhibited or unsure about the extent to which they were expected to, or would be supported to ‘give of themselves’ in their work.

Further, for new roles to be successfully adopted, organisational research suggests that the incumbents of new roles need to feel that they bring power to the team, as defined by a specific body of practice, especially if they are non-professionals [17]. It seems that Peer Workers in our study were not enabled to demonstrate the agency, or to directly influence the way the team works, that is understood to define an empowered actor in the workplace [43]. Studies of new HIV/ AIDS peer support educator roles [44] as well as other new roles in mental health services [45] are similarly indicative of how role adoption can be constrained where these conditions of shared expectation, role distinctiveness and enabled agency are not met.

Where Peer Workers in our study felt that they had not been accepted into the team as equal partners [46] – and especially where they felt they lacked the skills or training to engage in aspects of teamwork that required a specific clinical frame of reference – this dynamic also perpetuated a sense of not quite joining, or of remaining ‘other’ to the existing team [32]. The complexities of the Peer Worker identity were articulated; of neither being able to identify comfortably as staff or service user. Negative aspects of the ‘peer provider persona’ were also identified in a recent qualitative study of challenges faced by Peer Workers [35]. It might be that, in the absence of consensus on what constitutes peer practice, a distinctive sense of ‘being a Peer Worker’ with which to positively identify was somehow lacking.

### Strengths and limitations of the secondary analysis approach

Potential weaknesses in secondary analyses of qualitative data have been identified where there is not a good fit between data collected in the primary study and the questions asked of the data in the secondary analysis [47]. Our primary study was designed to elicit data on expectations and experiences of support for self-care provided by mental health services. We noted that many of our interviewees spoke about the role of users of those services employed as staff; the role was identified as a valuable aspect of support for self-care and the data to warrant closer analysis [25]. As such there was a reasonably good fit between primary data set and secondary analysis questions. However that fit was not even across the dataset. There was a relative lack of data from service user participants (compared to Peer Workers, non-peer staff and managers, from most of whom data was collected). A number of service user participants spoke about whether or not they felt ready to take on a Peer Worker role, but our interview schedule did not further elicit their views on their experiences of being supported by Peer Workers. This data would have strengthened our exploration of an emerging, distinctive body of peer practice. Future research in this area should be careful to address specific questions across a full range of relevant stakeholders. In addition, data informing some of our themes came predominantly from the London site, perhaps because that team had been most pro-active in trying to integrate the new role into the team. Again, a primary study around the introduction of Peer Worker roles in mental health services would set out to elicit similar data to investigate team building dynamics in a range of service specific contexts [14].

As strength, the robustness of our analysis was enhanced by the input of two service user researchers. New codes were created that foregrounded issues neglected in the preliminary framework, including staff retention of benevolent power as a constraint on the development of distinctive peer practice, and the experience of a conflicted Peer Worker identity. Issues such as flexibility and hierarchy were flagged as more than employment issues, characterising tension inherent in the Peer Worker experience. These issues helped shape the emergence of our meaningful themes. This cross-checking of the analysis by two researchers who were independent of the primary study, but who as service user researchers worked from a complementary standpoint was a strength of our approach, increasing the rigour of the methodological process and the explanatory power of the analysis.

## Conclusion

Interviewees in our study spoke positively about a number of benefits of Peer Worker roles. However, our analysis illustrated processes of introduction of new Peer Worker roles into mental health services that were characterised by lack of shared expectations of those roles, lack of consensus around what constitutes a distinctive body of peer practice and a lack of support enabling Peer Workers to bring that distinctiveness to the team. The role change literature we used to conceptually inform our analysis suggests that role adoption under these conditions will be constrained. It is important to note that all these initiatives were in an early, developmental stage, and that teams and their managers were not incognisant of these challenges and were working hard to address them. Learning from this study is suggestive of a number of important considerations for Peer Worker role development.

First, while informal or semi-formal approaches to recruitment straight from the service might be pragmatic while a service is in development these processes are unlikely to facilitate shared expectations of the role. The purpose and function of the role should be clearly articulated through the recruitment process, and the readiness of the potential Peer Worker to cope with the demands of the post properly assessed as part of selection. Dropout rates of potential Peer Workers during training or at the transition from training to working might also be reduced as a result.

Second, our study indicated that provision of flexible working arrangements for Peer Workers and benevolent, protective reinforcement of existing working practice within a team can militate against both the entry of Peer Workers as equal members of a team, and the emergence of a distinctive body of practice associated with the Peer Worker role. It seems likely that practices of peer working do not correspond neatly to existing clinical practice boundaries, especially in more clinically orientated services. All stakeholders to a service – service users, staff (peer and non-peer) and managers – need to share expectations of how Peer Workers are to engage with service users in order that understanding of how their work is to be boundaried is incorporated into job descriptions, training, supervision and so on. Broadly speaking, only when there is consensus about what constitutes a body of peer practice will Peer Workers have confidence to apply that practice in their work knowing that they will be supported and valued by colleagues and managers in doing so. While it seems likely that these expectations will be different in different services, our analysis suggests that it is important that organisations and teams do this careful thinking about how Peer Workers are expected to work prior to Peer Workers first coming into post. This might avoid the feelings of inequality, disempowerment, uncertainty about identity and of being under-supported that some of our Peer Worker interviewees reported.

Third, our study indicated that non-peer members of the team where not always aware of these discrepancies in expectation around the Peer Worker role; they believed that the team were working as equals when Peer Workers felt otherwise. Our analysis suggests that it is equally important that preparatory work – around developing consensus about the role and how Peer Workers practise – is undertaken with non-peer staff as it is with Peer Workers.

The role change literature cited above describes a ‘tipping point’ in the adoption of a new role at which a critical mass is achieved and a new role is institutionalised formally [17]. A critical mass of UK mental health service organisations may introduce Peer Worker roles. However there is a recent history in the UK of new mental health workforce roles that have not acquired the distinctiveness originally envisaged as they have been implemented across services nationally [45]. In the US the National Association of Peer Supporters is in the process of developing National Practice Standards that would define peer practice in terms of values, quality, ethical behaviour, job role and core competencies, potentially leading to the certification of the Peer Worker role as a profession [48]. Our data was not indicative of that degree of formality in the evolving roles we studied. However, our analysis does suggest that if the emergence of a distinctive body of peer practice is not adequately considered and supported in the UK, as integral to the development of new Peer Worker roles, then there is a risk that the potential impact of any emerging role is constrained and diluted.

## Abbreviations

NHS: National Health Service; WRAP®: Wellness Recovery Action Planning®; UK: United Kingdom; US: United States.

## Competing interests

The authors declare that they have no competing interests.

## Authors’ contributions

SGG was lead investigator on the primary study during which the data reported above were collected, and led on data analysis and drafting of this paper. CE participated in the design of the primary study and organisational aspects of data analysis. SLG participated in data analysis and drafting of this paper. KO participated in data analysis and drafting of this paper. CW participated in drafting of this paper. All authors read and approved the final manuscript.

## Pre-publication history

The pre-publication history for this paper can be accessed here:

http://www.biomedcentral.com/1472-6963/13/188/prepub

## Supplementary Material

Additional file 1: Table S3 Development of themes. Click here for file
