# Supplementary material for: Introducing peer worker roles into UK mental health service teams: a qualitative analysis of the organisational benefits and challenges
Source: BMC Health Serv Res. 2013 May 24;13:188. doi: 10.1186/1472-6963-13-188 (PMC3673834; doi:10.1186/1472-6963-13-188)
Supplement: Additional file 1: Table S3 — Development of themes. [file 1472-6963-13-188-S1.pdf]

Table 3. Development of themes.

Themes	Categories	Codes
Who becomes a Peer Worker, how and why?	1. Employment issues	Recruitment from the service<br>Recruitment process as a barrier<br>Ambivalence to recruitment process<br>Flexibility<br>Hierarchy<br>Pay & conditions<br>Voluntary to paid transition<br>Career development<br>Strategic support for peer work<br>Organisational costs & challenges<br>Deciding to be a Peer Worker (personal readiness; appropriateness of the work; motivation)<br>Anxieties (service users'; Peer Workers')
	2. Benefits for Peer Workers	Stepping stone/ enabler<br>Skills & confidence<br>Benefits to mental health
Being a Peer Worker: an experience of conflicted identity	3. Identity	Alternative to 'unwell' identity<br>Complex service user/ staff identity<br>'No-man's-land'
Building new teams	4. Peer Worker – staff relationship	Changing relationship/ power dynamics<br>Peer Worker-staff divide<br>Positive staff attitudes<br>Resistance<br>Forming a new team (hierarchy)<br>Retention of 'benevolent' power
	5. Training and support	Mental health of Peer Workers<br>Management of Peer Workers<br>On the job support (including peer support) & supervision<br>Content of training<br>Importance of 'hands on' training<br>Challenges of training
Challenging boundaries	6. Boundaries	Within staff team<br>Between Peer Worker & service users<br>Peer Worker's own boundaries<br>Practice boundaries
Is a body of Peer Practice emerging?	7. Role	Expectations of the Peer Worker role (service users'; Peer Workers'; staff's)<br>Responsibility<br>Professionalism<br>Capabilities<br>Relevance of past use of services<br>Relevance of past professional experience
	8. Benefits for team	Insight<br>Experience<br>Skills/ resource<br>Peer Workers as role models
